# Exploring Adolescents' Menstrual Hygiene Management Needs: Findings From Students in St. Louis, Missouri Charter Schools

**DOI:** 10.1111/psrh.70043

**Published:** 2025-11-18

**Authors:** Anne Sebert Kuhlmann, Kenneth Kibii, Kirstin Palovick, Cheleia Marshall

**Affiliations:** ^1^ College for Public Health & Social Justice Saint Louis University St. Louis Missouri USA

**Keywords:** menstrual health, menstrual hygiene, menstruation, period products, unmet needs

## Abstract

**Objective:**

To assess unmet menstrual hygiene needs and explore experiences with menstruation management among students at four charter high schools in the City of St. Louis, Missouri, United States in order to inform the development of future educational resources.

**Methods:**

Using a cross‐sectional design with mixed‐methods data collection, we collected quantitative data from 117 participants through anonymous, electronic surveys. We conducted four focus group discussions (FDGs), one at each high school, with 7–10 participants per group.

**Results:**

Among survey respondents, 41% were in 10th grade and a majority identified as non‐Hispanic Black (68%), with an average age of 16 years (15.6 ± 2.82 years). During the previous school year (2021–2022), over half of students (51%) were unable to access period products when needed at least once because they or their family could not afford them. The FDGs centered around four key domains of inquiry: (a) menstruation management‐related school resources, (b) menstrual symptoms and coping strategies, (c) menstrual hygiene education, and (d) menstrual cycle tracking strategies.

**Conclusions:**

Our findings highlight how essential it is to address the issue of unmet needs for menstrual products and hygiene management to ensure all students who experience menstruation can access period products, minimize menstruation‐related absenteeism, and promote the overall well‐being of adolescent students.

## 1 Introduction

Globally, menstrual health is a key issue for human rights and gender equality [1, 2]. Menstrual health encompasses the provision of accurate information to menstruating individuals, access to menstrual products, and hygienic washing facilities [3]. The phrase “period poverty” refers to not having access to the resources, including the education, products, privacy, and hygiene materials, necessary to manage menstruation hygienically [4].

Though research has revealed period poverty and gaps in menstrual hygiene management (MHM) in low and middle‐income countries, there is still limited knowledge of the needs and experiences of adolescents in the United States (US) [5]. A small, qualitative, pilot study in North Dakota found students had challenges accessing period products at school [6]. Another qualitative study of adolescents across three cities in the US reported gaps and challenges with puberty and menstruation education in schools [7]. Moreover, there is evidence that period poverty has increased in the US over recent years, especially among young adults [8, 9].

Around the world, girls with poor MHM may miss school regularly, drop out of school altogether, and/or experience more vaginal and urinary tract infections [10, 11, 12]. In the US, approximately 13% of survey respondents reported missing at least one day of school while in high school because they did not have access to menstrual hygiene products when needed [5]. In another study, nearly 69% of respondents missed at least one day of school per month for any reasons associated with their periods, including heavy flow, odor, and pain/cramping [13]. Between 17% and 34% of female students reported missing school because they did not have an adequate supply of period products when needed [13, 14]. Among a sample of US high school students who menstruate, 65% reported they sometimes used toilet paper or paper towels, with the percentage increasing to 80% when menstruation started at school [5]. The inability to afford period products and manage menstruation can create challenges both for attending and fully engaging in school over many years.

Adequate menstrual hygiene goes beyond access to period products to also include access to privacy where one can change products comfortably, soap and water for washing as necessary, and the hygienic disposal of used sanitary materials [15]. It involves creating a stigma‐free environment where individuals can make informed decisions and fully participate in all aspects of life without discrimination or exclusion based on menstruation [16]. In the US, adolescents report discussing menstruation most commonly with their mothers, at school, and with their healthcare providers. Many felt that the current timing of menstrual health education in schools did not prepare them for menarche and that the education provided was inadequate [17].

Recent results from a national cohort study indicate that the average age of menarche in the US is decreasing and is now less than 12 years old [18]. Furthermore, the proportion of individuals experiencing early (< 11 years old) and very early (< 9 years old) menarche has nearly doubled since the 1950s [18]. Therefore, it is more crucial than ever to begin educating students early about proper menstrual hygiene.

Over the past few years, several US states have passed policies aimed at addressing period poverty. However, critics note that these policies fail to address MHM more broadly in schools in tandem with their requirements around product provision [19]. A review of state school health education standards throughout the US found that most states do not include any requirements around menstruation management or period products in their curricula [20]. Other research has found that nearly half of high school students want more information/education on managing their periods and caring for their personal hygiene during menstruation [13].

Building on these findings, it is important to explore further students' menstrual hygiene needs and experiences managing menstruation within the school context. Here, we assessed menstrual hygiene needs among high school students and explored their experiences managing menstruation at school using a mixed‐methods approach with the goal of informing the development of future educational materials for students in these schools around menstruation and MHM.

## 2 Methods

To assess and document the impact of unmet menstrual hygiene needs among students and their experiences managing menstruation at school we employed a mixed‐method, cross‐sectional study design, utilizing both anonymous, electronic surveys and in‐person focus group discussions (FDGs) during the 2022–2023 academic year. Building on existing relationships, four of the six charter high schools operating in St. Louis that year chose to participate in the study. Eligibility criteria for both the surveys and the focus groups included being enrolled at one of the four participating charter schools, being in grades 9–11 and 14–17 years old, and having reached menarche. Because part of the purpose was to inform the development of future educational materials, we focused on students who still had one or more years remaining at the schools. Saint Louis University's Institutional Review Board approved this study.

### 2.1 Electronic Surveys

We created the electronic survey in Qualtrics XM. We collected responses from September through October 2022. The survey asked students questions about experiences during both the previous school year (2021–2022) and the then‐current school year (2022–2023).

Prior to the survey distribution, the schools sent a consent form with a parental opt‐out option home with all students. No parents opted out of the survey. In Missouri, students are enrolled as male or female, so schools filtered for female students and then sent an email that included a link to an anonymous, self‐administered, electronic survey to students in grades 9–11, just over 500 female students total. All four schools have a non‐academic advisory/counseling period during the week. The schools distributed the survey link during these times, so as not to disrupt an academic class period. While students were given time during their advisory/counseling period to complete the survey, the link remained open for approximately 6 weeks so that students could complete the survey at a time and location of their choosing. The schools sent two reminder emails to encourage participation.

We provided an informed assent statement on the first screen of the survey. In total, 150 students started the survey. Of these, 117 participants (approximately 23% of those to whom the survey was distributed) met the inclusion criteria of having reached menarche, being in the 9th to 11th grade, and being between 14 and 17 years of age. All 117 were included in the analysis. After survey completion, a separate pop‐up survey allowed students to indicate if they wanted the incentive of a $5 pharmacy retail store gift card, which we then distributed through the schools.

The survey included basic demographic questions—current age, age at menarche, grade, and race/ethnicity—plus questions about period products, managing and consequences of menstruation, menstrual hygiene resources at school, menstrual hygiene education, and recommendations to help address menstrual hygiene needs. We analyzed survey data using IBM SPSS Statistics 29, calculating frequencies for all variables. We tested the association between missing school and using a period tracking app, current grade level, and years since menarche using logistic regression. We calculated odds ratios (ORs) using 95% confidence intervals (95% CI) with statistical significance determined with a *p*‐value < 0.05.

### 2.2 Focus Group Discussions

After preliminary analysis of the survey data, we conducted in‐person FGDs to garner a more in‐depth understanding of the menstruation experiences of high school students, especially within their school context. We recruited a convenience sample through the schools using announcements and flyers. The FGDs took place between December 2022 and February 2023 at each of the four charter schools with a facilitator and note‐taker leading each group. Prior to the FGDs, schools sent home another recruitment statement with a parental opt‐out option. At one school, four parents returned the opt‐out form. The school ensured those students did not participate in an FGD. Students indicated their interest in participating either through their counselor/social worker or the front office, depending on the school.

The FGDs consisted of 7 to 10 participants per session. The facilitator overviewed the research aim and reviewed the assent form. All students orally assented to participate and also to audio recording and transcription. We designed the FGD guides to delve more deeply into the topics of the survey questions. The moderator utilized a semi‐structured guide asking about (1) school resources, (2) symptoms and coping strategies, (3) menstrual hygiene education, and (4) menstrual cycle tracking strategies. Each FGD session lasted between 45 min and 1 h. Participants received a $15 gift card to a local pharmacy retail store.

The principal investigator (ASK) facilitated the focus groups and trained the note‐takers (KP & CM) prior to the start of data collection. The facilitator and note‐takers were all women who experience menstruation, navigate MHM themselves, and who have experience working with adolescents. The note‐takers during the FGDs (KP & CM) reviewed and edited the automated transcripts. The principal investigator (ASK) approved the final transcripts prior to coding. A preliminary codebook was created a priori with anticipated themes based on the domains of inquiry in the FGD guides; emergent codes were added during analysis. Data were coded and analyzed using Dedoose version 9.0.54. Two co‐authors (KP & CM) individually coded all the transcripts and then compared them for consistency to produce the final, coded transcripts. Inter‐rater reliability was tested in Dedoose; the pooled Cohen's kappa of 0.89 indicated strong agreement between coders [21]. Any discrepancies were addressed in discussion with the principal investigator (ASK).

## 3 Results

### 3.1 Electronic Surveys

Of the 117 surveys, 41% of the participants were in 10th grade, and a majority identified as non‐Hispanic Black (68%), with an average age of 16 years old (15.6 ± 2.82 years). On average, respondents reached menarche around 11 years old (11.39 ± 1.5) (Table 1).

**TABLE 1:** Demographic and menstruation‐related background characteristics of survey respondents (*n* = 117).

Variable	Frequency (%)
Age (in years) (*m*, ±SD)	15.6 **±** 2.82
Grade level	
9th grade	34 (29.1)
10th grade	48 (41.0)
11th grade	35 (29.9)
Race/ethnicity (*n* = 108)	
Non‐hispanic black	73 (67.6)
Non‐hispanic white	21 (19.4)
Hispanic/other	14 (13.0)
Age at first menstrual period (in years) (*m*, ±SD)	11.39 **±** 1.5
Students who track their periods (% Yes) (*n* = 106)	103 (97.2)
Types of period products used (% Yes)^a^	
Disposable pads (*n* = 106)	85 (80.2)
Disposable liners (*n* = 101)	14 (13.9)
Disposable tampons (*n* = 102)	15 (14.7)
Reusable period underwear (*n* = 98)	13 (13.3)
Reusable/washable cloth pads (*n* = 101)	7 (6.9)
Menstrual cup (*n* = 99)	1 (1.0)
Needed period products at least once during the *last* school year (2021–2022) but they or their family did not have enough money to buy them (*n* = 94)	48 (51.1)
Used homemade pads/tampons or got period products from elsewhere because they or their family did not have enough money to buy them during the *current* school year (2022–2023) (% yes) (*n* = 93)	52 (55.9)
Main source of information about periods and personal hygiene during menstruation (*n* = 92)	
Family member or friend	55 (59.8)
Community organization	2 (2.2)
School	3 (3.3)
Self‐taught (e.g., internet, book, reading the product package)	32 (34.8)
Would like more information/education on managing their periods and caring for their personal hygiene during their periods (*n* = 91)	
Yes	41 (45.1)
No	31 (34.1)
Don't know	19 (20.9)

^a^ Multiple response options possible.

Nearly all survey respondents (97%) track their period in some form—using a period tracking app (49%), an electronic or handwritten calendar (39%), a notebook or sticky notes (18%), a personal diary (17%), or a notes application on their phones (7%). Disposable pads were the most commonly used products among students (80%), with a smaller percentage using disposable tampons (15%) or liners (14%).

During the previous school year (2021–2022), over half of the students (51%) had been unable to access period products at least once when needed because they or their family could not afford them. To manage their menstruation, these students reported asking friends and family for products (68%), receiving products from the school nurse (53%), making homemade period products (36%), asking a teacher or school staff member (34%) or elsewhere in school (23%), or getting donated products from an outside organization (12%). Participants primarily learned about menstruation from family or friends (60%), followed by being self‐taught (35%) through looking up information online, reading books, or referring to the product packaging (Table 1). Only 3% of participants indicated school as their main source of menstruation information. Nearly half of the students, though, wanted more education and information on how to manage their menstruation.

Most students were aware of the resources available to them at school. The majority reported they could access period products in the school nurse's office (72%), followed by teachers (54%) and counselors/social workers (39%) (Table 2). Just over 10% of students reported they could access period products in the school restroom, either freely available (12%) or in vending machines (9%). However, over 18% of students did not know where they could obtain period products at school.

**TABLE 2:** Student experiences managing menstruation at school (*n* = 117).

Variable	Frequency (%)
Places at school where students can access period products	
Nurse's office (*n* = 106)	
Yes	76 (71.7)
Don't know	19 (17.9)
Administrative office (*n* = 99)	
Yes	15 (15.2)
Don't know	61 (61.6)
Teacher(s) (*n* = 101)	
Yes	54 (53.5)
Don't know	32 (31.7)
Counselor/social worker (*n* = 100)	
Yes	39 (39.0)
Don't Know	51 (51.0)
Bathroom(s), freely available (*n* = 102)	
Yes	12 (11.8)
Don't know	24 (23.5)
Bathroom(s), vending machines (*n* = 101)	
Yes	9 (8.9)
Don't Know	27 (26.7)
Issues due to menstruation in the *last* school year (2021–2022) (% Yes)^a^	
Missed school or been absent (*n* = 93)	43 (46.2)
Left early or have come late (*n* = 93)	46 (49.5)
Disciplined at school (e.g., penalized for not dressing out in gym, being late to class, not participating in class) (*n* = 92)	31 (33.7)
Avoided going to the restroom to change products at school (*n* = 92)	38 (41.3)
Had trouble accessing products while school was out of session over extended breaks, e.g., winter break, spring break, or summer (*n* = 92)	9 (9.8)
Missed school at least one day each month in the *last* school year (2021–2022) (% Yes)^a^	
Pain or cramping (*n* = 93)	58 (62.4)
Heavy bleeding (*n* = 92)	51 (55.4)
A bad odor (*n* = 93)	40 (43.0)
Not having adequate products to manage period (*n* = 93)	21 (22.6)
Missed school so far in *current* school year (2022–2023) (% Yes)^a^	
Pain or cramping (*n* = 93)	35 (37.7)
Heavy bleeding (*n* = 92)	32 (34.8)
A bad odor (*n* = 92)	16 (17.4)
Not having adequate products to manage period (*n* = 90)	13 (14.4)

^a^ Multiple response options possible.

Despite access to these resources at school, many students still reported missing school due to their periods. During the prior school year (2021–2022), almost half of the respondents reported missing school due to their period (46%) (Table 2). Factors contributing to missing school include pain or cramping (62%), heavy bleeding (55%), bad odor (43%), and not having an adequate supply of period products (23%). Furthermore, half of the participants had either come to school late or left early because of their period.

Even if students did attend the full school day during their period, many reported being unable to participate fully. Over one‐third of menstruating students (34%) reported getting in trouble at school for reasons related to their periods, for example, not dressing out during gym class, arriving late to class, or not participating in class (Table 2). Moreover, 41% of students reported avoiding going to the restroom during the school day to change their products when needed due to privacy concerns. Beyond the school year, around 10% of students reported having difficulty accessing period products during extended school breaks.

Out of 104 students who responded to questions about managing their menstrual pain/cramps, 46% used over‐the‐counter pain medications during every menstrual cycle and 18% used them occasionally. Similarly, 38% used heating pads every menstrual cycle, while 65% used them occasionally.

There was no statistically significant association between missing school at least once a month for any menstruation‐related reason and the predictors of using a period tracking app, grade level, or years since menarche (*p* = 0.69).

### 3.2 Focus Group Discussion Findings

Our domains of inquiry for the FGDs centered around four topics: menstruation‐related school resources, menstrual symptoms and coping strategies, menstrual hygiene education and support for managing menstruation, and menstrual cycle tracking strategies. Within these domains, students indicated questions they had about menstruation, menstrual hygiene, and period products. These questions both reflected their lack of specific knowledge around menstruation as well as what topics they would like to learn more about through future educational materials. They also made recommendations for what would help them to manage their periods more effectively and how their schools could better support them in doing so. A summary of each domain, with supporting quotes, is presented below.

#### 3.2.1 Menstruation‐Related School Resources

Most FGD participants indicated that their school has period products available in the nurse's office. Some students also reported that teachers and staff members have products in their rooms that students can access. According to students, the school nurses mostly supply disposable pads. Several students were dissatisfied with the type and variety of pads provided, saying the pads available were not absorbent enough for them. As one student at Charter School A put it, “[the nurse] only gives out the small thin pads like what am I supposed to do with that? I'm over here heavily bleeding and like what am I supposed to do with that?”

Students would like their schools to offer a wider variety of period products to support the diverse needs of menstruating students. For example, respondents who experience heavier periods desire thicker pads, while those who have lighter periods or who participate in athletics want access to thin pads that are more comfortable and discreet. A student at Charter School D reflected, “I feel like they should have a variety of sizes. … I'm like, I can't wear this super big one, cause I'm dancing. I have on tights and a leotard. […] I feel like they should have a variety of sizes because if you want a pad, you should be able to get a pad. It should never be poking out so you're embarrassed.”

Several students reported negative experiences managing menstruation at school, specifically around bathroom cleanliness, privacy, and accessibility during the school day. Students noted that the bathrooms were frequently unclean, as their classmates do not dispose of menstrual products appropriately or clean up after themselves. Several students commented on non‐functional soap dispensers.

[Charter School D] “The bathrooms here are absolutely disgusting. Especially the female restrooms. [Classmates] will literally not clean up after themselves at all whenever they're on their period. […] And also, the trash bins, like, sometimes it will be overflowing with period products and stuff. And, like, the toilets; they'll just be having blood stains and a bunch of other stuff on them like where it's just repulsive. And just can't even use a bathroom.”

Furthermore, some students felt teachers did not support them with respect to managing menstruation and menstrual symptoms. As one student at Charter School D put it, “I feel like a lot of the other female teachers, or not even just female but male as well, could put more support into the fact that teenage students have their period. Sometimes they're heavy, sometimes they're very light. I feel like that more of the faculty could, like, provide like pads and tampons, especially those who are less fortunate and stuff.” Teachers must balance individual student's needs with their overall classroom management and education responsibilities.

[Charter School D] “[T]he other day, I like, had just started my period, and I bled completely through, like, blood was everywhere. I didn't know I had my period. So, I was going to my next class, and I knew I had to go to the bathroom because something wasn't right, and the teacher in there, he was […]being, like, so rude about it. I like kept telling him that I need to go to the bath‐, the restroom. And he would not write me a pass to let me go or anything, and he told me he was gonna write me up. And he wrote me up”.

#### 3.2.2 Menstrual Symptoms and Coping Strategies

Menstrual pain during the school day was common for many students every menstrual cycle and frequently made it difficult to participate in class. Students cope with their menstrual pain in a variety of ways; however, as different strategies work for different individuals. Students at Charter School A expressed a range of coping strategies from, “I put my head down and go to sleep” to “I usually fall sleep in class because of the pain” to “I'll thug it out but if I'm in too much pain I'll go downstairs, go to the nurse ask her if I can lay down for a few minutes, then I'll be good.” Students reported some coping strategies being harder at school due to the lack of freedom during school hours.

A few students report extreme physical symptoms during menstruation, including severe cramps, body aches, nausea, and vomiting. Examples from Charter School A included, “I'm thinking I'm having a miscarriage (amount of blood)”, “I feel like I'm dying”, and “I feel like I'm getting murdered. Like a whole murder.” A student from Charter School C said, “It'll be so bad because whenever I'm on (my period) I throw up.”

Several students also described emotional and psychological discomfort while menstruating. The emotional changes that can accompany the menstruation cycle, for example, pre‐menstrual syndrome (PMS) were discussed explicitly and implicitly during all the FDGs. Comments from two students in Charter School C reflected these discussions: “I have pain, but I'd be mad”, and “…Almost every girl knows that feeling when you're on your period […] like you, you're like self‐conscious feeling like everyone around you like, do they notice? Can they smell me? Like, all this stuff. But the thing is, it's like it's embarrassing like uncontrollable.”

Acknowledging schools' rules, many students described wanting various products to help with the management of menstruation, especially pain and cramping. Several participants described how the schools offered only limited options for those experiencing menstrual cramps. Although many students described what they needed to manage menstrual cramps, such as over‐the‐counter pain medication and heating pads, access to those during the school day can be limited. As a student in Charter School A explained, “For us to get [over‐the‐counter pain medication], we have to call our parents for [the nurse] to verify that you can get it.” In addition, some girls noted that putting their heads down in class to cope with the pain may result in negative academic coursework and consequences during class.

In all four schools, participants reported missing school due to their periods, including leaving school early or not coming to school at all. Missing school was a common coping strategy for students to manage their periods. Pain and heavy bleeding were common reasons given by students for missing school due to their periods.

#### 3.2.3 Menstrual Hygiene Education

Participants expressed a wide range of knowledge, attitudes, and beliefs about menstruation. They obtained information about menstruation from a variety of sources, including friends, family, school, and, especially, social media and the internet. Importantly, they mentioned numerous questions they would like answered about period products, the biology and physiology of the female and male body, and management of menstrual hygiene and menstrual symptoms. Students at Charter School A asked, “What is an abnormal amount of bleeding?” and “Yeah, pain is normal, but is throwing up and crying normal?”, while a student at Charter School C asked, “Why do women have periods in the first place, what is the biological purpose?”. These questions reflect a general lack of knowledge about menstruation and suggest opportunities for future educational resources.

A student at Charter School C reflected on not being taught how to manage her personal hygiene during menstruation, “I'm not even gonna lie to you, like period blood does not smell good. So, like I was never taught like how you're supposed to like keep yourself, like, freshened up and stuff like that.”

Several students expressed frustration at the absence of a comprehensive sex education curriculum at their school and wished adults, including their parents, were more comfortable discussing sexual and reproductive health with them. They also expressed concerns about being taught about menstruation management by men or older individuals. For example, a student from Charter School C said, “I feel like people don't talk about it enough with very young children, especially females, who are kind of coming close to that age, or something. And I feel like it should be something that people are comfortable talking about.” Another student from the same school shared, “But like, for […] a class, like a sexual education class, or like anything like that um. I feel like you have to have certain credentials for it. I don't want to be taught by a man.”

#### 3.2.4 Menstrual Cycle Tracking Strategies

Several participants had attempted to use period tracking apps before, but they felt the apps were unreliable and inaccurate. Participants also had questions about how the apps work and how the apps can tell the user when to expect their period or when it is late. These comments and questions about how such apps work reflect a general lack of knowledge and understanding about menstruation and the menstrual cycle. For example, a student in Charter School A asked, “How do [the period tracking apps] be knowing if you don't come on? Like, there was one time I didn't come on that whole month, and it says that I'm late. Like how does it know? I didn't even put it in there.” Another student in Charter School A asked a similar question, “Like how does [the period tracking app] know ‘oh it is going to be this day’ and how does it track it?”

## 4 Discussion

These data were collected in the context of Missouri initiating a new feminine hygiene grant program for schools through the Department of Elementary and Secondary Education. Through the program, public and charter schools are eligible to receive reimbursement for period products and other designated menstrual hygiene‐related supplies. The reimbursement amount varies by school using a formula based on the number of female students enrolled in grades 6–12 plus a modifier for overall census poverty level in the district [22]. Therefore, Missouri makes some reimbursement funding available for schools to provide period products but does not require that they must do so. In addition, Missouri, like most states in the US, requires health education around puberty, pregnancy prevention, abstinence, and prevention of sexually transmitted infections but does not have any educational standards for public and charter schools around menstruation management or period products [20].

The participating schools all make period products available for their students, despite not being required to do so. Students expressed both a significant desire for and use of these school resources, particularly from the nurse's office (72%). The students' reported use of school resources to access period products was similar to previous studies [13, 14], and reinforces the importance of these products being freely available through schools. While students desired a wider variety of period products available in their schools to meet their differing needs, they did utilize what resources are currently available. More schools consistently providing free access to a variety of period products for their students and states providing the financial resources to help them do so should help address a critical issue in education in the US that has been noted for over a decade now [15].

Even within the contexts of schools making period products freely available to students and the state providing reimbursement funding for schools to do so, over 50% of students reported experiencing period poverty at some point during the prior school year, and 22% reported missing school the prior school year because they lacked period products. These percentages are similar to previous studies in other schools [5, 13, 14]. Combined with the large percentage of students who reported accessing products at school, these findings reinforce how vitally important period products are to have freely available in schools and suggest we should consider additional ways for students in need to access period products.

Importantly, other menstruation‐related reasons for missing school, such as pain and cramping or heavy flow, account for the majority of menstruation‐related school absences and are consistent with percentages reported in previous studies [13, 14]. Schools must consider how they can help students manage menstruation more generally in order to mitigate the impact on attendance. Given that challenges managing menstruation account for a large proportion of absenteeism related to menstruation and yet few states have any health education standards around menstruation management [20], US states, including Missouri, should consider expanding their health education standards to include menstruation management, use of various period products, and personal hygiene during menstruation.

Students in the FGDs expanded on how menstruation affects their attendance at school and their attention to and concentration in classes. These findings are consistent with findings from other studies among US students [23]. Together, these studies highlight how addressing the effect of menstruation on education will require a comprehensive approach and must go beyond just making period products available in schools.

Beyond a wider variety of period products, students expressed a desire for more school resources to help manage menstrual pain and symptoms and to receive more education around menstruation and menstrual hygiene. The FGDs also highlighted complaints about menstrual symptoms interfering with daily activities, including school. On the one hand, studies have reported that school nurses self‐report feeling competent enough to support girls with menstrual pain and identify if the pain is normal or may be indicators of other gynecological problems [24]. On the other hand, while participants' symptoms may be normal, research has found that among adolescents with dysmenorrhea or chronic pelvic pain the overall prevalence of endometriosis is about 62% [25]. Pain management resources are currently less available to students at school than period products and the level of pain described by some students may indicate other gynecological problems. It is important to expand education for both students and school nurses around what is “normal” versus abnormal related to menstrual flow, pain, cramping, and other symptoms so that students can get connected to outside health resources if their symptoms warrant it.

Nearly half of the survey participants indicated that they would like more information on menstruation and hygiene during menstruation. However, just a few students stated that they had received information like this from school. Other studies have reported similar desires for improved menstrual hygiene education among both adolescents [26] and adults [27]. Likewise, teachers and school nurses [28, 29] have also expressed a desire for additional training around providing menstrual education. School nurses report that their students need more education around MHM [29]. Some research has uncovered tensions, however, between the authority of home and school when teaching children about menstruation and puberty. Previous research also identified issues with the delivery of information, such as insufficient and uninteresting material available to teachers [7]. Our findings suggest that considering who delivers the education to students around menstruation is also important as men and older individuals may be seen as less authoritative on the topic. These are challenges schools will need help navigating to reduce menstruation‐related absenteeism.

The questions asked by students in the FGDs suggest they have very limited knowledge overall about menstruation, how and why it occurs, and how it affects one's body. While previous research has indicated that knowledge varies tremendously by whether a student has had a period [9], the questions expressed in our study all originated from students who menstruate. Other research suggests that high school students from both rural [30] and urban [31] areas of the country also have questions about products, odor, and managing hygiene during menstruation The questions expressed by participants in our study indicate a strong need for more education around menstruation and menstrual hygiene. The questions are also providing a starting point for us to develop additional educational resources for students in these schools.

Over a third of students responding to our survey indicated they had been disciplined at school for reasons connected to their periods. In the FGDs, students described specific situations where they were punished or felt ostracized by teachers for what they were experiencing with their periods. Teachers in a study elsewhere in the US expressed similar concerns about students missing class due to menstruation, menstruation being a distraction from the classroom, and perceptions that most menstruating students' experiences at school were unpleasant, stressful, embarrassing, and centered around hiding [31]. Our findings, in conjunction with those of earlier studies [23, 31], document the feelings of embarrassment that adolescents have about menstruation. They also highlight the need for teachers and school staff to be educated about the menstrual health needs of students, how menstruation may influence students' attention and focus during class, and how they can support students.

Almost all students reported tracking their periods, and nearly half use period tracking apps on their phones to do so. However, comments during the FGDs suggest that students have a lot of questions about these apps plus have limited knowledge about how the apps calculate and track one's menstrual cycle based on users' data. The students' questions about how the apps work also highlight the variability they experience in their menstrual cycles. This is common, especially for those who have recently reached menarche. Results from a national cohort study in the US indicate that for nearly half of individuals it can take up to 2 years post‐menarche for menstrual cycles to normalize and become predictable [17], so students' questions may reflect the challenges they face in tracking and predicting their cycles. This irregularity and unpredictability of students' menstrual cycles further highlight the importance of schools always having products freely available, of students knowing where and how to access these products, and being comfortable doing so, when needed. Our results suggest that expanded menstrual hygiene education for students should include an introduction to tracking one's menstrual cycle, how apps or other techniques may help, and what the challenges may be. Such education should also include an understanding that non‐regular periods are “normal”, especially during the first few years post‐menarche.

### 4.1 Limitations

While this is one of the first mixed‐methods studies among US high school students around menstrual hygiene needs and experiences managing menstruation at school, it does have important limitations. First, our self‐reported data on school absences due to menstruation may be subject to recall bias. However, the consistency in findings between this and other studies [5, 13, 14] strengthens our confidence in these results. Furthermore, we relied on self‐reported data because schools only track “illness” generically for absences and do not record whether menstruation contributes to those absences. Second, even though the survey data collection was anonymous, studies have documented stigma associated with menstruation and period poverty [19, 32, 33] that may result in underreporting challenges accessing products and managing menstrual hygiene. Third, our study was limited to charter schools in the City of St. Louis because it was designed to inform the creation of additional educational resources for these schools. While these schools serve a similar student population as the City's public schools in terms of racial and household economic status [34], there may be important differences between students who choose to attend charter schools and those who do not. Therefore, future studies should explore these themes more broadly in larger school districts across the country. Fourth, our inclusion criteria focused on students who had reached menarche, irrespective of gender identity. Because Missouri students are enrolled as male or female, filtering for female students may have excluded a few students enrolled as male who menstruate. Future research should explore the experiences of menstruation management at school specifically among students who may not identify as female. Finally, students who may have missed school due to their periods on the day of the FGD at their school did not have the opportunity to participate and share their experiences.

Despite these limitations, as a team of researchers who manage menstruation themselves and have experience working with adolescents, we believe that we were able to foster a safe, open environment for students to discuss their experiences with menstruation. The conversations in all the FGDs were robust and animated. Students asked many questions about reproductive physiology, menstruation, menstrual symptoms, and menstruation management that they were genuinely interested in exploring. Our findings provide a richer understanding of these students' experiences and challenges managing their menstrual hygiene within their school environment.

### 4.2 Implications

Schools and their staff, particularly school nurses, play a significant role in addressing students' menstrual hygiene needs and helping them navigate menstruation to minimize the impact on their daily lives, including attendance at and attention to school. Our findings reinforce the need for free access to period products at schools as many students rely on these resources. States, including Missouri, should ensure and expand funding for schools to provide period products in schools along with auxiliary resources such as pain management, personal hygiene products, and spare underwear and clothing. Additionally, schools should ensure that students are aware of these resources and that they are easily accessible. Furthermore, schools need to educate faculty and staff regarding how menstruation may affect not only students' attendance but also their concentration and focus during class and share ways that they can be supportive of such students. Finally, states, like Missouri, need to expand their health education standards to include a focus on menstruation, the menstrual cycle, MHM, and period products as even students who have been menstruating for several years have significant questions around these topics. In the near term, we are developing and pilot testing infographics for these schools based on the students' questions around menstruation.

## 5 Conclusions

It is essential to address menstrual hygiene needs and support students in managing menstruation to minimize the impact of menstruation on their daily lives, especially on their education. With the average age of menarche decreasing in the US and more students experiencing early and very early menarche, we must prioritize access to period products and menstrual hygiene education in schools in order to promote students' well‐being. Policies should go beyond just the provision of period products and focus on addressing the overall context of managing menstruation at school.
